# A three-dimensional nano-network WO_3_/F-TiO_2_-{001} heterojunction constructed with OH-TiOF_2_ as the precursor and its efficient degradation of methylene blue

**DOI:** 10.1039/d1ra04809k

**Published:** 2021-07-29

**Authors:** Chentao Hou, Jing Hao

**Affiliations:** Department of Environmental Engineering, Xi'an University of Science and Technology Xi'an 710054 China houct@xust.edu.cn

## Abstract

In this study, three-dimensional nested WO_3_/F-TiO_2_-{001} photocatalysts with different WO_3_ loadings were prepared by a hydrothermal process and used to degrade methylene blue (MB). The photocatalysts with various ratios of WO_3_ to OH-TiOF_2_ can be transformed into a three-dimensional network WO_3_/F-TiO_2_ hetero-structure with {001} surface exposure. The results showed that the composite catalyst with 5% WO_3_, denoted as FWT5, had the best comprehensive degradation effect. FWT5 has a limited band gap of 2.9 eV, which can be used as an advanced photocatalyst to respond to sunlight and degrade MB. The average pore diameter of the composite catalyst is 10.3 nm, and the multi-point specific surface area is 56 m^2^ g^−1^. Compared with pure TiOF_2_, the average pore size of the composite catalyst decreased by 8.44 nm and the specific surface area increased by 51.2 m^2^ g^−1^, which provides a larger contact space for the catalytic components and pollutants. Moreover, TiO_2_ on the {001} surface has higher photocatalytic activity and methylene blue can be better degraded. Under the irradiation of 0.03 g FWT5 composite catalyst with a simulated solar light source for 2 h, the degradation rate of 10 mg L^−1^ methylene blue can reach 82.9%. The trapping experiment showed that photo-generated holes were the principal functional component of WO_3_/F-TiO_2_-{001} photo-catalysis, which could capture OH^−^ and form hydroxyl radical (˙OH) and improved the photocatalytic degradation performance. Kinetic studies show that the photocatalytic degradation of MB fits with the quasi-first order kinetic model.

## Abstract 2

A new type of WO_3_/F-TiO_2_-{001} heterostructure semiconductor material with a three-dimensional network structure was successfully prepared by the hydrothermal method.

## Graphical abstract

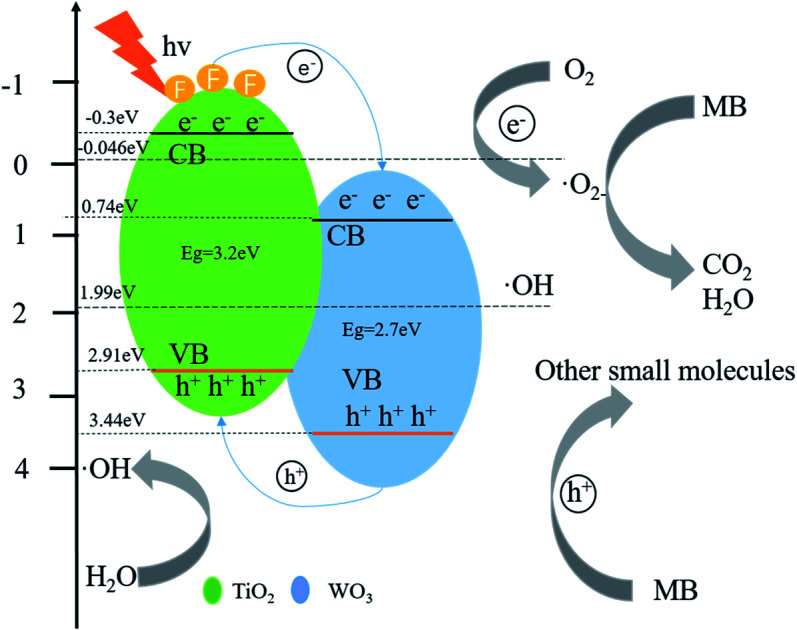

## 1. Introduction

Over the past decades, with the rapid development of the world's industry and economy, human beings are extremely dependent on various chemicals, which threaten our living environment. The main pollutants in wastewater are organic colored dyes with relatively stable performance and complex molecular structures. Among them, MB is one of the most common dyes in wastewater, which can be harmful to human health, and can lead to eye burning, dyspnea, accelerated heart-rate, emesis, sweatiness, insanity, nausea and methemoglobinemia.^1,2^ Due to the difficulty of biodegradation, it is very difficult to remove this organic matter from wastewater, which requires enormous resources. The commonly used methods to remove the dye from wastewater include precipitation, ion exchange, solvent extraction, coagulation flocculation, filtration, electrochemistry, and adsorption. However, these methods require high energy consumption, high costs, low concentration, and have insufficient degradation rate.^3,4^ Among other technologies for the treatment of wastewater, photocatalytic technology has attracted more attention due to its environmental friendliness, mild reaction conditions, low price, clean process and high efficiency, especially in its application to the degradation of MB.^5^

Recently, a variety of photocatalytic materials have been developed to degrade organic pollutants, such as Ag-based nanocomposites, alveolate Cu_2−*x*_Se microsheets, MoS nanosheets, MoS_2_/sepiolite, and MoS_2_/sepiolite.^6–10^ In this paper, TiO_2_ is used, as one of the most common semiconductor photocatalysts, which has the advantages of usability, corrosion resistance, nontoxicity and good chemical stability.^11–14^ However, TiO_2_ has a wide band gap of 3.0–3.2 eV, which causes it to respond only under ultraviolet radiation, and its photocarriers recombine rapidly, which seriously hinders its application and development in practice.^15,16^ In order to solve this problem, many researchers used metal doping,^17–19^ nonmetal doping,^20^ precious metal deposition,^21,22^*etc.* The introduction of dopants can effectively reduce the band gap width of TiO_2_, which makes TiO_2_ absorb in the visible region, and increases the internal electron acceptor, thus slowing down the carrier recombination rate.

In addition, the surface properties of TiO_2_ can be greatly enhanced by adding F ions. Tong *et al.*^23^ discovered that the photocatalytic performance of powdered TiO_2_ or TiO_2_ film doped with F ions was obviously superior to that of pure TiO_2_, the crystal form of TiO_2_ is changed by addition of F ions, and the crystallinity of anatase is increased, which led to the formation of the high-energy {001} surface. Therefore, the photocatalytic performance is improved.^24^ In another study, Han *et al.*^25^ developed TiO_2_ nanosheets with 89% TiO_2_{001} crystal faces by adding hydrofluoric acid to tetra-butyl titanate under hydrothermal treatment, which showed that the photocatalytic performance of F-TiO_2_ nanosheets was significantly better than that of P25. It should be noted that TiO_2_ can form TiOF_2_ when the F ion concentration is high^26^ and interestingly, TiOF_2_ can be converted back to TiO_2_ under different conditions.^27^ Zhang *et al.*^28,29^ studied the formation process of TiO_2_-{001} nanosheets by adding different amounts of hydrofluoric acid and controlling different reaction times and temperatures. Shi *et al.*^30^ studied the conversion process of cubic TiOF_2_ to TiO_2_-{001/101} at different calcination temperatures, and put forward a probable conversion mechanism of TiOF_2_ to TiO_2_-{001/101}. Zhang *et al.*^31^ found that TiOF_2_ acts as an unstable intermediate during the transformation of TiO_2_ nanoparticles into TiO_2_-{001} nanosheets. TiO_2_-{001} nanosheets due to their higher hydrogen production,^32–35^ and photocatalytic reaction can efficiently degrade MB and RhB. The synergistic effect of TiO_2_ and TiOF_2_ nano-particles further improves the photocatalytic activity of TiO_2_-{001} nanosheets. In our pervious study we have found that TiO_2_-{001/101} nanosheets can be derived from TiOF_2_ under hydrothermal conditions.

Since TiO_2_ has a large band gap of 3.2 eV and can only absorb ultraviolet radiation, it is necessary to synthesize a photocatalyst that can absorb visible light. Another type of semiconductor oxidizer is tungsten trioxide (WO_3_) with a narrow band gap (2.4–2.8 eV), and due to its low energy band gap, it can respond under visible light. Therefore, due to the enhanced ability of visible light photons, WO_3_ shows significant photocatalytic activity,^36^ and has attracted more attention since it is non-toxic, economical, and has promising characteristics.^37^

In order to separate the charges effectively, researchers combined TiO_2_ with a semiconductor metal oxide with a suitable band gap, which resulted in a reduction of recombination rate of electrons–holes, and improvement of the photocatalytic performance of TiO_2_. For example, TiO_2_ is combined with CeO_*x*_,^38^ V_2_O_5_ (ref. 39) and WO_3_ (ref. 40) which effectively separates the charges. Recently, the application of WO_3_/TiO_2_ composites and the heterojunction of WO_3_/TiO_2_ composites are widely studied by many researchers.^41–43^ The WO_3_–TiO_2_ nanocomposite is an energy storage photocatalyst, which can store electrons generated under light, and release these electrons in the absence of light, which occurs in electron-mediated reactions.^44,45^ For instance, Yang *et al.*^41^ used a WO_3−*x*_/TiO_2_ film as a new photoanode which reduced CO_2_ to form formic acid through photocatalysis. Wang *et al.*^42^ found that the prepared WO_3_/TiO_2_ nanotube composite film showed a significant increase in the degradation rate of volatile organic compounds under a photocatalytic degradation process.

In our previous research, Hou *et al.*^46^ washed the prepared TiOF_2_ with an alkali which significantly improved the catalytic performance of the catalysts, since TiOF_2_ has more O–H associations on its surface. Therefore, in this paper, we combined WO_3_ with OH-TiOF_2_ at high temperature and high pressure. In this research, after one-step hydrothermal treatment, WO_3_ with different proportions and OH-TiOF_2_ transformed into a three-dimensional network WO_3_/F-TiO_2_-{001} hetero-structure. According to FTIR measurements there were more associated O–H on the surface of TiO_2_, which could significantly enhance the catalytic activity of TiO_2_ for MB oxidation under visible light. This catalyst has good photocatalytic activity for methylene blue. In addition, more importantly, the photocatalyst has excellent stability and reusability, which has a good prospect for the practical application of dye wastewater degradation. Interestingly we have found that there are few published research studies using WO_3_ with OH-TiOF_2_ as a photocatalyst for degradation of dyes.

## 2. Experimental

### 2.1 Synthesis of composite photocatalysts

Preparation of OH-TiOF_2_: first 34 mL of butyl titanate was magnetically stirred, and 30 mL of glacial acetic acid was added dropwise slowly into butyrate titanate. Then 12.5 mL of hydrofluoric acid (HF) was added to the mixture and continuously stirred at room temperature for 0.5 h. Following that, the obtained mixture was placed in a PTFE lined reactor for 15 h at 160 °C. Next, the reactor was cooled at room temperature, and the solid product was obtained by centrifugation and washed with absolute ethanol and ultrapure water three times respectively. Finally, the product was obtained by drying in an oven at 90 °C. Then, 6 g of the prepared TiOF_2_ powder was added into a 100 mL glass beaker, 10 mol L^−1^ sodium hydroxide solution was added over the powder, and then the mixture was stirred at a constant speed at room temperature for 2 h; afterward, the mixture was centrifuged and washed with anhydrous ethanol plus ultrapure water three times respectively. Then the product (OH-TiOF_2_) was dried at 80 °C in an oven and ground before use.

Preparation of the WO_3_/F-TiO_2_-{001} composite catalyst: different dosages (0.1056 g; 0.1759 g; 0.2815 g; 0.3519 g; 0.62084 g; 1.5521 g; 3.58 g) of sodium tungstate dihydrate were dissolved in 30 mL ultrapure water, and magnetically stirred for 10 min at low speed until the sodium tungstate completely dissolved. After that, 20 mL of 2 mol L^−1^ HCl solution was added to the above mixture and stirred at 30 °C for 2 h. Then 1 g of OH-TiOF_2_ powder was added and continuously stirred for 30 min until the OH-TiOF_2_ powder entirely dissolved. Next, the solution was transferred to a reactor and placed in a drying oven at 160 °C for 4 h. After the reactor cooled at room temperature, the mixture was centrifuged and the product was obtained by washing the centrifuged sediment with ultrapure water 3 times. The WO_3_/F-TiO_2_-{001} composite catalyst was obtained by drying at 100 °C in an oven and then ground and denoted as FWT3, FWT5, FWT8, FWT10, FWT20, FWT50, and FWT100 before use.

### 2.2 Characterization

An X-ray diffractometer (XRD, p-XD-2, China) was used to observe the crystal characteristics of the catalyst under a 36 kV, 20 mA, Cu-kα X-ray source (*λ* = 0.15418 nm). The element distribution and surface morphology of the samples were analyzed by EDS and SEM (Japan JSM7500F). A nitrogen adsorption and desorption specific surface area analyzer (BET, micrometrics ASAP2020, USA) was used to determine the specific surface area and porosity of the catalyst using BET and BJH methods. The chemical valence states of the sample were analyzed by X-ray photoelectron spectroscopy (XPS). Absorption characteristics were measured over the 200–800 nm scanning range using a UV-Vis DRS (Shimadzu, UV-2600, Japan). Fluorescence spectrometry (PL, Shimadsu-rf-6000, Japan) was used to measure the electron–hole recombination at an excitation wavelength of 300 nm in a scanning range of 250–650 nm. The measurement range of Fourier transform infrared spectroscopy (FT-IR) is 4000–400 cm^−1^, and it is carried out in the KBr particles using a Nicolet IS5 spectrometer of the United States to identify the functional groups on the catalyst surface.

### 2.3 Photocatalysis experiment

Herein, photocatalytic degradation of methylene blue was used to evaluate the activity of the catalyst. 100 mL of methylene blue solution with a concentration of 10 mg L^−1^ was added into a reaction tube and then 30 mg of semiconductor photocatalytic material was added. A 300 W xenon lamp was used to simulate sunlight for the photocatalytic degradation of dye solution. Then the lamp was turned off and kept for 30 min to reach the adsorption equilibrium; next, the light was turned on and 7 mL of the reaction solution was taken out every 15 min to measure the simulated pollutants. The supernatant was centrifuged at a high speed (10 000 rpm), and then measured using a UV-Vis spectrophotometer with a wavelength of 662 nm. The absorbance of the dye solution was measured before and after the reaction, and the concentration of pollutants before and after the reaction was determined by the standard curve, and the dye removal rate was calculated according to the change of the concentration. The photocatalytic degradation of dye solution and antibiotic solution was investigated. Methylene blue was used as the simulated degradation product to investigate the active substances which play a major role in the WO_3_/F-TiO_2_-{001} photocatalytic reaction. For degradation of MB, 30 mg of WO_3_/F-TiO_2_-{001} composite catalyst with the optimal photocatalytic performance ratio of 5%, and 100 mL of methylene blue solution with a concentration of 10 mg L^−1^ were studied under simulated sunlight and the experiment was repeated three times.

The three active substances (hydroxyl radicals, holes, and superoxide radicals) were captured in degradation processes. For this purpose, 0.3 mmol of *tert*-butanol (*t*-BuOH) was used to take hydroxyl radicals, following that, 0.3 mmol of methanol (MT) was applied to capture holes and in the third step, 0.1 mmol of benzoquinone was added to catch the superoxide radicals. The rest of the condition was kept unchanged through the entire experiment. Consequently, the photocatalyst was separated from the dye solution in order to study its durability and reusability.

## 3. Experimental results and discussion

### 3.1 Characterization of the catalyst

#### 3.1.1 Morphological analysis of the catalyst

**Fig. 1:**
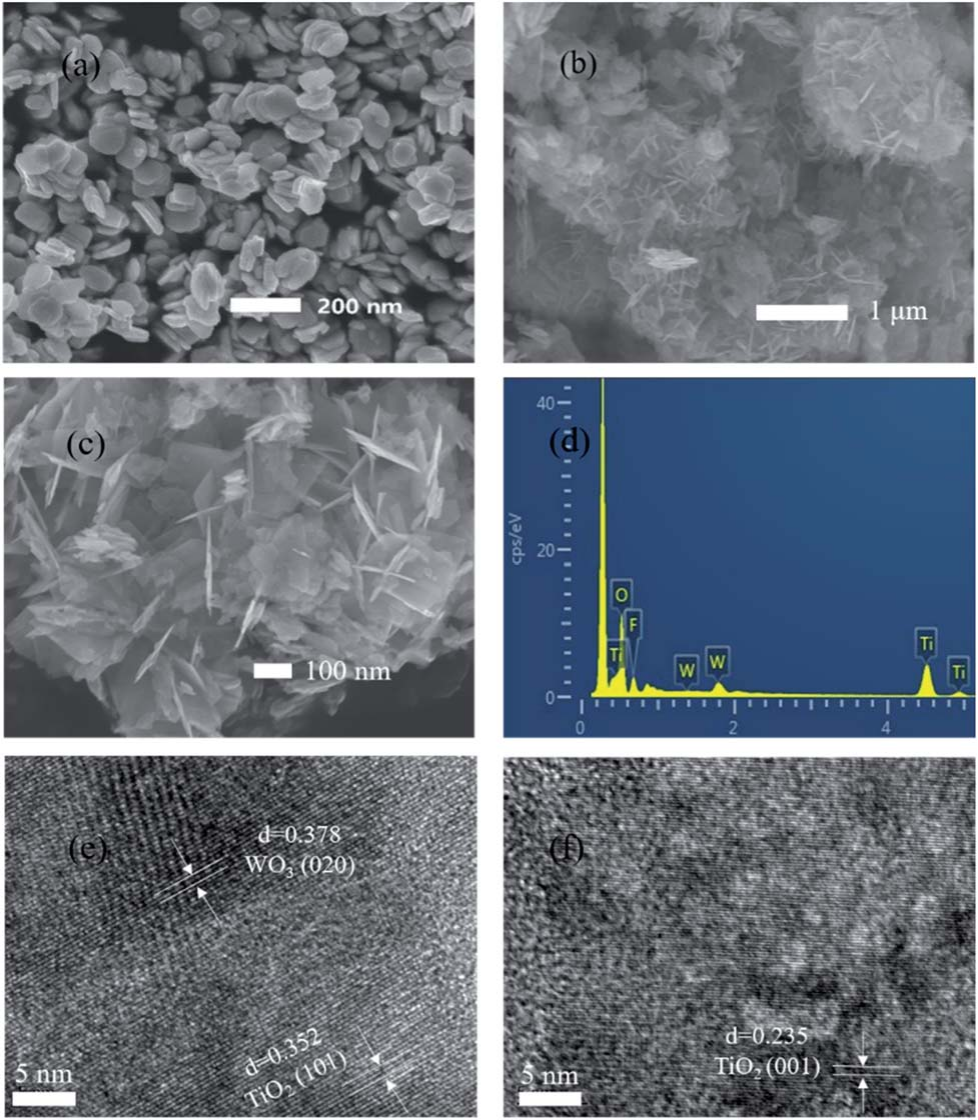
SEM images of (a) WO_3_, (b) OH-TiOF_2_, and (c) WO_3_ : Ti = 5%, (d) EDS spectrum of WO_3_ : Ti = 5%, and (e) and (f) HRTEM images of WO_3_ : Ti = 5%.

The morphology and composition of WO_3_ (a), OH-TiOF_2_ (b) and WO_3_ : Ti = 5% composites (c) were analyzed by SEM. The sizes of the composite catalyst and alkali-washed TiOF_2_ are 100 nm and 1 μm, respectively, the scale of WO_3_ is 200 nm, and the space between grains is 200–400 nm, with a uniform size and good dispersion. From the comparison of (a)–(c), the large sheet should be TiO_2_, and the small sheet is WO_3_. In Fig. 1b, the alkali washed TiOF_2_ shows a more complex network shape, and the alkali washed TiOF_2_ shows phase assembly along a certain direction. The net structure makes the photocatalytic surface area larger. From the FWT5 (c) diagram, WO_3_ and TiO_2_ are mutually doped and finally present a three-dimensional net-like structure. Due to the relatively low proportion of doped WO_3_, we can observe the scaly shape of TiO_2_ with a non-uniform surface size and the lamellar WO_3_ hidden under the TiO_2_ surface. The network structure formed in this way provides a more catalytic area for photocatalysis. Fig. 1d shows the EDS spectrum of the WO_3_/F-TiO_2_ composite catalyst. Samples containing mainly O, F, W and Ti show four characteristic peaks, and their contents (weight percentage) are 41.64%, 11.21%, 5.67% and 41.48%, respectively. Furthermore, no excess impurity peak was seen, and the morphology of the prepared sample was analyzed using a high resolution transmission electron microscope (HRTEM), as shown in (e) and (f). The lattice fringes are 0.352 nm and 0.235 nm respectively, corresponding to the lattice distances of the anatase type TiO_2_(101) plane and (001) plane.^47,48^ It can be seen that the (001) crystal is exposed on the surface of F-TiO_2_.

#### 3.1.2 Crystal structure

**Fig. 2:**
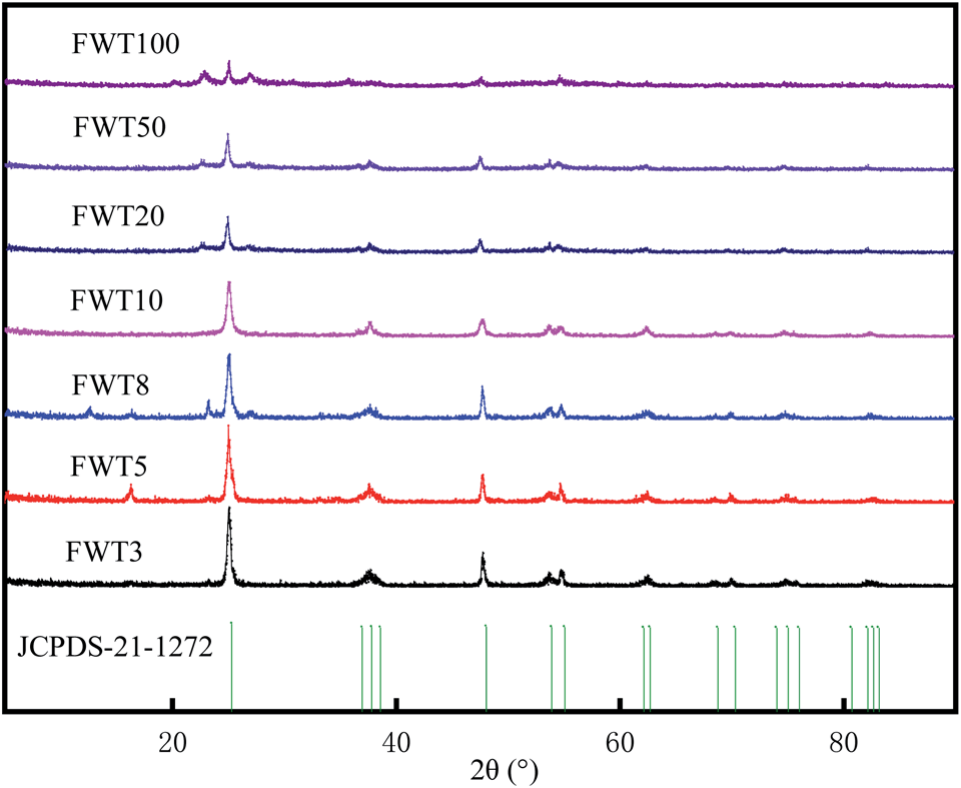
XRD spectra of TiO_2_, WO_3_ and WO_3_/F-TiO_2_-{001}catalysts with different ratios of W to Ti.

The XRD patterns of each sample are shown in Fig. 2. Compared with the JCPDS standard card (no. 211272), the 2*θ* values of XRD curves of seven kinds of photocatalysts with different composite ratios were 25.32°, 38°, 47.96°, 53.98° and 62.9° respectively, corresponding to anatase type titanium dioxide {101}, {004}, {200}, {211}, and {204} crystal faces. This indicates that in these photocatalysts doped with different proportions of tungsten trioxide, only anatase phase exists, and no other phase of titanium dioxide exists. From the diffraction patterns of FWT8 and the percentage less than 8%, it was observed that the peak position of WO_3_ is not obvious at 23.1°, but it also increases with the increase of content, which is due to the low WO_3_ content. The diffraction pattern of WO_3_ : TiO_2_ = 100%, 50% and 20% composite catalysts shows that the peak at 23.1° is enhanced and expanded, which corresponds to the main peak of WO_3_ in the standard card of XRD (no. 321395). However, with the increase of WO_3_ content, when the 2*θ* values are 25.32°, 38°, 47.96°, 53.98° and 62.9°, the characteristic peak intensity of TiO_2_ gradually weakens.

#### 3.1.3 BET specific surface area and pore size

**Fig. 3:**
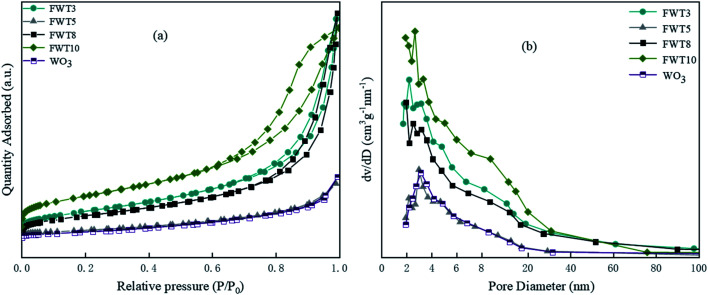
(a) Nitrogen adsorption/desorption isotherm of FWT3, FWT5, FWT8, FWT10 and WO_3_. (b) The pore size distribution corresponding to each catalyst.

The pore size distribution and specific surface area of the composite materials with different proportions were analyzed by nitrogen adsorption desorption technology. From Fig. 3, we can see the N_2_ adsorption desorption curves of catalysts with different ratios and the corresponding BJH pore size distribution. We can see from Fig. 3a that the catalysts we studied exhibit a typical type IV N_2_ adsorption and desorption isotherm, and the H3 type hysteresis loops of all catalysts show obvious opening, which indicates that the catalysts have mesopore formation.^49^ The adsorption capacity of the catalyst increases with the increase of *P*/*P*_0_. In addition, according to Table 1, the average pore size of the composite catalyst decreased from 18.74 nm to 10.30 nm, and the specific surface area increased from 4.80 m^2^ g^−1^ to 56.00 m^2^ g^−1^. Compared with WO_3_ and other composite catalysts, FWT5 has a larger specific surface area than WO_3_, FWT3 and FWT8 and a larger mesoporous size relative to FWT10. In order to examine the expansion of FWT5 surface area, BET and FESEM figures were combined together; it is speculated that the increase of specific surface area of FWT5 is due to the loose accumulation of particles during the process of TiO_2_ covering the WO_3_ surface, which forms many gaps and increases the surface area of the catalyst. As a result, more active sites are exposed to the composite photocatalyst to generate sufficient electron–hole pairs with REDOX capacity, and the active factor of the catalyst can come into contact with MB molecules more fully, thus improving the photocatalytic activity of the material. In addition, mesopores also play an important role in the photocatalytic activity of the material, as the catalyst with a developed pore structure can make light refraction and scattering in the material many times, and stimulate more active sites. Hence, the catalyst can further stimulate more electron–hole pairs, resulting in enhancement of the photocatalytic activity.^50,51^ Moreover, larger mesopores also facilitate fast mass transport, which improved the catalytic performance.^52–55^ The smaller the average pore size, the larger the specific surface area, the richer the catalytic active sites of the catalyst, and the faster the mass transfer of the reactants and products which lead to improvement of the photocatalytic activity. Fig. 3b shows the pore size distribution diagram of the catalysts. It can be seen from the figure that the pore diameter of the prepared catalysts is mostly concentrated in 2–6 nm, which is the distribution range of the catalyst particle size. Based on the figure, FWT5 has the largest pore size and adsorption capacity.

**Table 1:** Characteristics of samples analyzed by N_2_ adsorption desorption experiments

Sample	Specific surface area (m^2^ g^−1^)	Pore volume (cm^3^ (STP) g^−1^)	Average pore size (nm)
WO_3_	12.06	0.056	15.81
TiOF_2_	4.80	0.02	18.74
FWT3	49.21	0.17	14.00
FWT5	56.00	0.16	10.30
FWT8	41.06	0.18	17.23
FWT10	75.04	0.17	8.80

#### 3.1.4 The light absorption characteristics

**Fig. 4:**
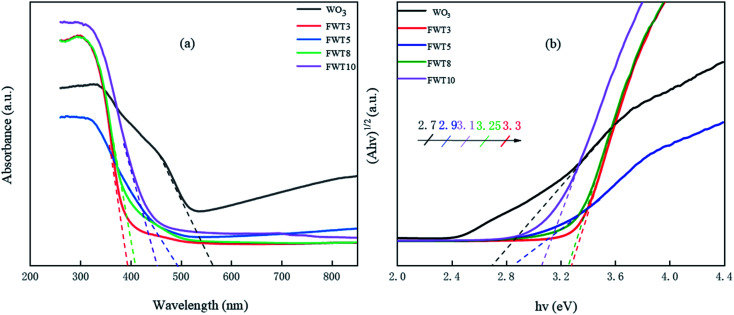
(a) UV-Vis absorption spectroscopy and (b) the corresponding band gap energy of WO_3_ and FWT*X* (*X* = 3, 5, 8, and 10).

The light absorption properties of the composite materials were characterized by ultraviolet-visible diffuse reflectance absorption spectroscopy (UV-Vis DRS). It can be clearly seen from Fig. 4a that different photo-catalysts and the same catalyst with different recombination ratios have different absorption properties in the UV region. The light absorption capacity increases in the order of FWT5 < WO_3_ < FWT3 < FWT8 < FWT10 in the ultraviolet region. The absorption threshold of the FWT5 photocatalyst at 491 nm corresponds to an energy band gap of 2.9 eV. It can be seen that the FWT5 composite catalyst can greatly improve the utilization of simulated sunlight. The red shift phenomenon of the composite catalyst in varying degrees indicates that its response to simulated sunlight is significantly enhanced, which might be due to the formation of more electron hole pairs.^56^ The smaller the band gap, the wider the response range.^57^ Therefore, it is easier for the FWT5 composite photocatalyst to use visible light. The band gap energy (*E*_g_) of the FWT3, FWT5, FWT8, FWT10 and WO_3_ was calculated according to the Tauc plots. Based on Fig. 7b, the band gap energies of FWT5, FWT3, FWT8, FWT10 and WO_3_ are calculated to be 2.9 eV, 3.3 eV, 3.25 eV, 3.1 eV and 2.7 eV, respectively.

**Fig. 5:**
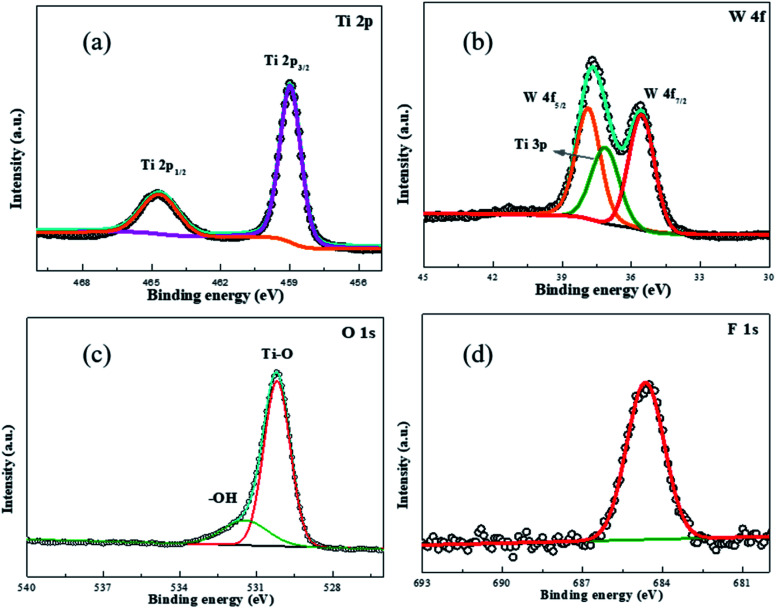
Fine spectrum of FWT5L: (a) Ti 2p, (b) W 4f, (c) O 1s, and (d) F 1s.

**Fig. 6:**
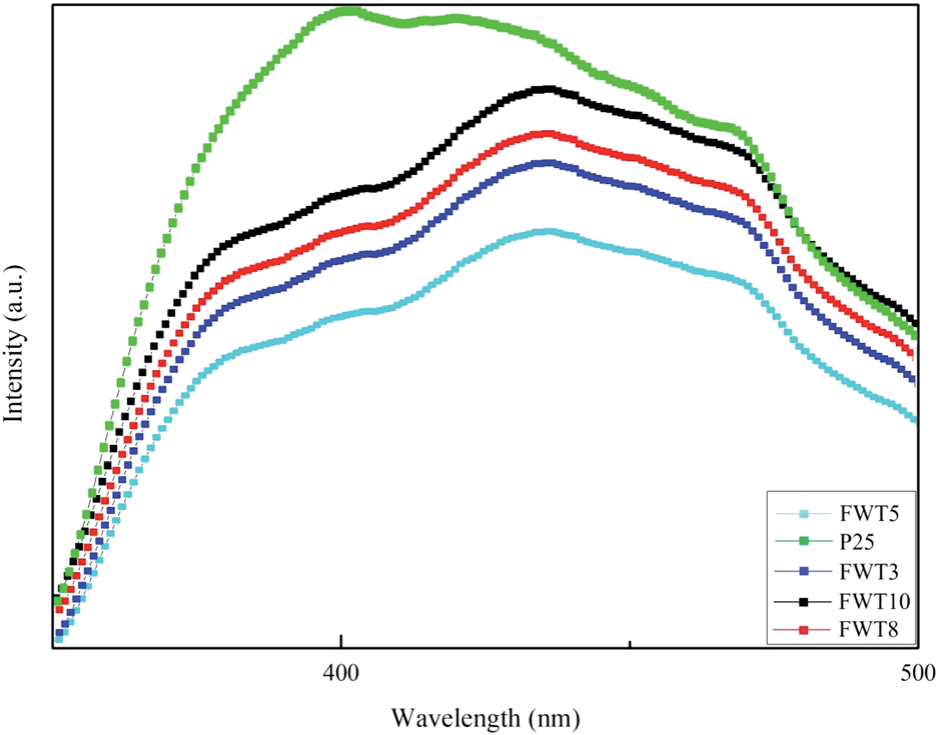
Band gap width of TiO_2_ and FWT5 composite catalysts.

**Fig. 7:**
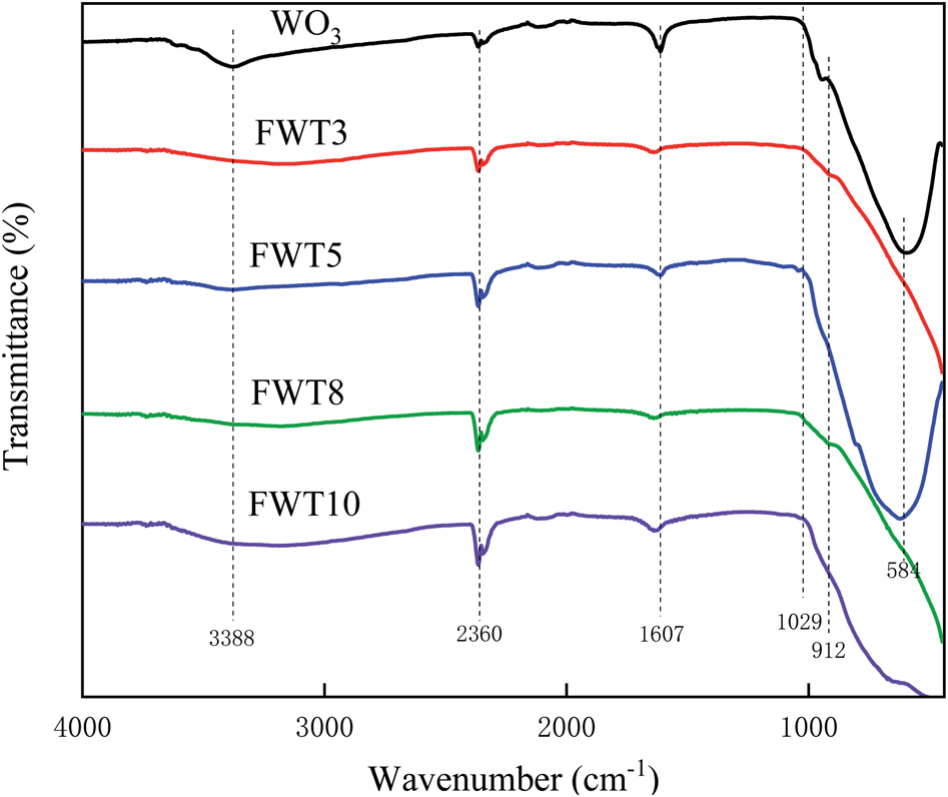
FT-IR spectra of WO_3_ and W : Ti = *x* : 100 (*x* = 3, 5, 8, and 10) with different Ti(*x*) molar ratios.

#### 3.1.5 Surface element analysis

The elemental composition and valence states on the surface of the sample at a depth of 3–5 nm were investigated by X-ray photoelectron spectroscopy (XPS). All data were corrected by the binding energy (284.6 eV) of C 1s. Fig. 5a is the fitting spectrum of Ti 2p; the peaks at 459.0 eV and 464.7 eV belong to the 2p_3/2_ and 2p_1/2_ signals of Ti^4+^ in TiO_2_ respectively.^58^Fig. 5b shows the fitting spectrum of W 2p. The peaks at 35.5 eV and 37.8 eV belong to the 4f_7/2_ and 4f_5/2_ signals of W^6+^ in WO_3_, and the peaks at 37.1 eV belong to the signals of Ti^4+^ 3p.^59^Fig. 5c is the fitting spectrum of O 1s, the peak at 530.2 eV in TiO_2_ and WO_3_ belongs to the lattice oxygen signal, and the peak at 531.5 eV is attributed to –OH adsorbed on the material surface.^60^ From Fig. 5d we can get the fitting spectrum of F 1s, and the signal at 684.6 eV belongs to the signal of F 1s.^61^ The results of XPS analysis showed that the WO_3_/TiO_2_ composite catalyst was successfully prepared, but F ions were doped and –OH was adsorbed on the surface of the material after alkali washing. This also explains why TiO_2_ disappears and transforms into TiO_2_ after alkali washing. Although the presence of WO_3_ could not be seen from the SEM of 5% of the composites, it may be due to the low content of WO_3_, but the existence of F was confirmed by XPS, and the transformation of TiO_2_ to TiO_2_ could be seen by XRD.

#### 3.1.6 Electron hole recombination degree

Photoluminescence (PL) measurements can effectively detect the recombination process of photo-generated carriers and their separation efficiency; the increase in luminescence intensity is caused by the increase in the recombination rate of photo-generated electron–hole pairs. Fig. 6 shows the photo emission spectra of P25 and FWT composite catalysts. We can observe from Fig. 6 that the lowest peak intensity of FWT5 is much lower than that of the P25 sample, and the fluorescence peak intensity is the lowest, which indicates that it is more difficult to capture photo-generated electrons, which leads to a reduction in the recombination of holes and photo-generated electrons, thereby improving the photocatalytic activity. The broad emission band (350–450 nm) of the peak located in the ultraviolet region is linked with the titanate group between the defects trapping free excitons, which is caused by the emission of bound excitons.^62^ Due to the existence of oxygen vacancies,^63^ a wide band (450–500 nm) will be emitted in the visible region, which means that the composite catalyst has more oxygen vacancies than single TiO_2_.

#### 3.1.7 FT-IR

FT-IR was used to analyze and characterize the structure of the composite catalyst. It can be seen from Fig. 7 that the corresponding absorption peaks were generated near 1607 cm^−1^ and 3388 cm^−1^ due to the bending vibration and stretching of the water molecules and O–H attached to the surface of the catalyst.^64–66^ The band at 2300 cm^−1^ corresponds to the vibration of atmospheric carbon dioxide,^67^ and absorption peaks near 584 cm^−1^ are caused by Ti–O–Ti vibration stretching.^68^ The catalyst only produced a slight absorption peak near 912 cm^−1^, which was caused by the stretching vibration of the Ti–F bond formed by F-TiO_2_.^69^

### 3.2 Degradation effect on dye wastewater

#### 3.2.1 Degradation effect of the catalyst

**Fig. 8:**
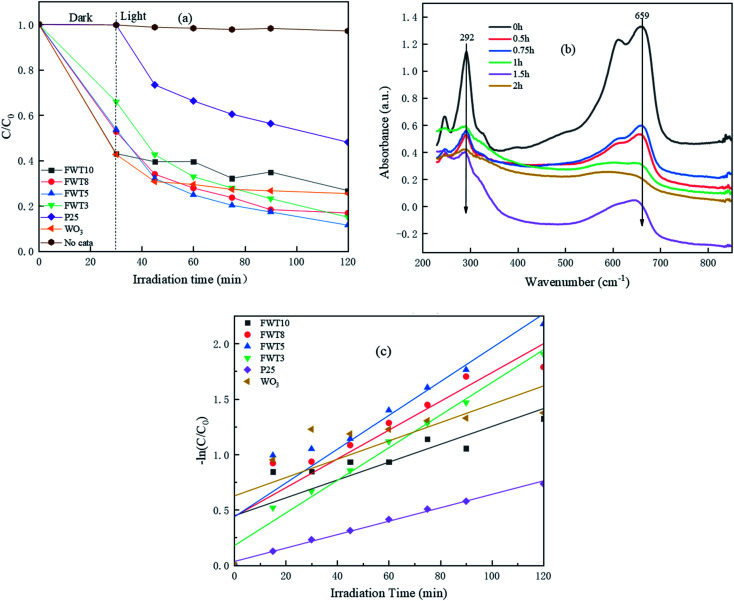
(a) Photo-degradation of MB by different samples under simulated sunlight (the dosage of the catalyst is 30 mg); (b) UV-Vis absorption spectrum of FWT5; (c) kinetic curve transformation of photo-degradation of MB solution with different samples.

In order to study the photocatalytic performance of the samples, the typical printing and dyeing wastewater methylene blue in sewage was taken as the research object, and the solar photocatalytic degradation test was simulated. Fig. 8 shows the degradation curves of methylene blue by photo-catalysts under simulated sunlight. As can be seen from Fig. 8a, when the system reaches the equilibrium of adsorption and desorption, WO_3_/F-TiO_2_-{001} has good adsorption performance, and changes with the ratio of W : Ti. This is due to the irregular accumulation of WO_3_ particles on the surface of TiO_2_, resulting in an increase in specific surface area and porosity, and the highly exposed {001} surface, making the FWT10 sample have the strongest MB adsorption capacity. This is also consistent with the obtained BET characterization results. Although the adsorption capacity of FWT3 for MB is weaker than that of FWT10, its total removal rate (78.4%) is higher than that of FWT10 (70.6%). This phenomenon shows that the photocatalyst's ability to adsorb MB cannot determine its ability to degrade MB. The total degradation rate of FWT5 to MB reached 82.9% within two hours under simulated sunlight, which was higher than the 74% of WO_3_ and 62.2% of commercially available P25.

Fig. 8b shows the variation of the MB absorption spectrum with light time in the presence of WO_3_/F-TiO_2_-{001} under visible light irradiation. At 664 nm (the characteristic absorption peak of MB), the peak intensity of MB showed an obvious decreasing trend, indicating that MB was photodegraded under visible light irradiation. Moreover, the photocatalytic degradation effect of the catalyst with the best composite ratio (FWT5) on MB at a wavelength of 664 nm almost completely disappeared after 2 h of irradiation. At the same time, no new absorption bands were observed during the photodegradation process, which confirms the complete photodegradation of the MB aqueous solution.

By studying the kinetic model of WO_3_/F-TiO_2_-{001} photocatalytic degradation of MB, the photocatalytic performance of the sample was further evaluated. It can be seen from Fig. 8c and Table 2 that the *R*^2^ of the composite photocatalyst is close to 1, which indicates that the process of degrading methylene blue meets the first-order reaction kinetic model, and the reaction constant *K* value (0.0126) of the 5% composite catalyst is slightly higher than that of the others. The *K* value of the composite catalyst is twice that of the single catalyst (0.0054), indicating that the degradation rate of the 5% composite catalyst has increased.

**Table 2:** *R* ^2^ and *K* values of the first order kinetic model fitted by different catalysts

	FWT3	FWT5	FWT8	FWT10	P25	WO_3_
First order dynamics *R*^2^	0.9856	0.9983	0.987	0.9518	0.9955	0.9752
First order kinetic constant *K*	0.0124	0.0126	0.0111	0.0051	0.0064	0.0054

#### 3.2.2. Analysis of the WO_3_/F-TiO_2_ photocatalytic mechanism

**Fig. 9:**
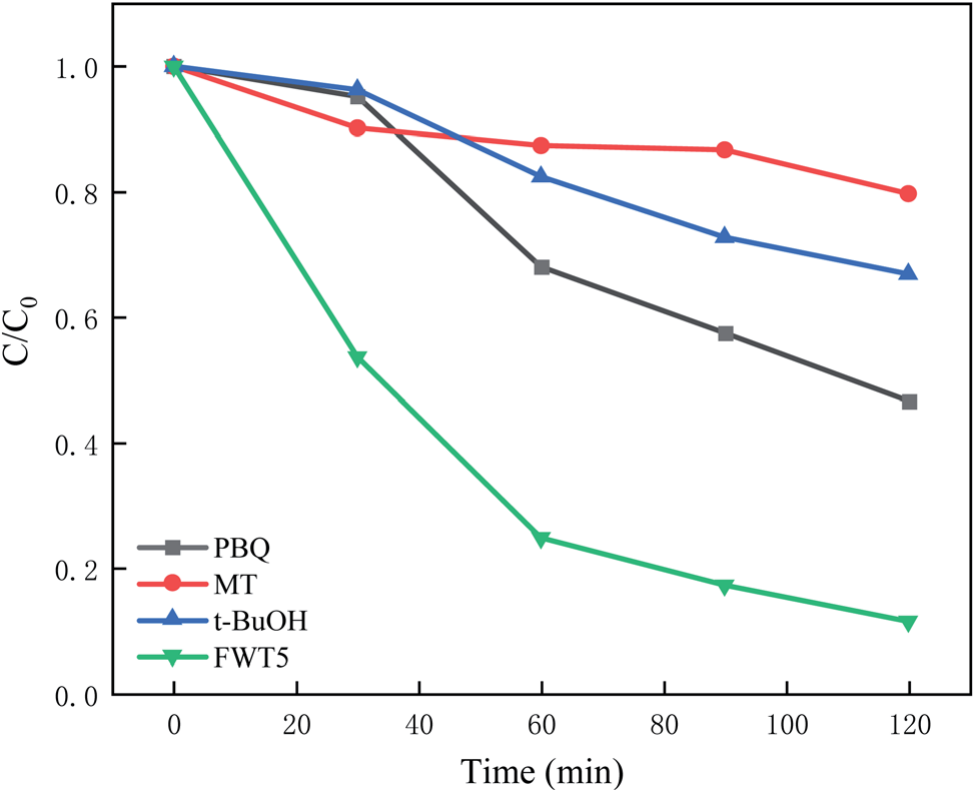
Free scavenging experiment of FWT5 (40 mg of catalyst and 3 mmol L^−1^ scavenger were added).

Generally, there are three active substances in the process of photocatalytic degradation of MB solution: hydroxyl radicals (˙OH), superoxide radicals (˙O_2_^−^) and holes (H^+^). Due to this phenomenon, we introduced trapping agents such as 1,4-benzoquinone (PBQ, ˙O^2−^),^70^ methanol (MT, h^+^), *tert-*butyl alcohol (*t*-BuOH, ˙OH)^71^ and so on for capturing free radicals in the current experiments. Thus, we can identify the active components which have more effect on the degradation process of MB. The conditions of capture experiment and photocatalytic experiment are the same. From Fig. 9, in the process of dark reaction, the addition of scavengers has no obvious degradation effect on MB, and different scavengers combined with the FWT5 show different adsorption effects.

Adding *tert*-butanol and methanol has little effect on the photocatalytic degradation of MB solution, and the degradation rate decreased by 60% and 70% respectively. The results showed that holes and hydroxyl radicals determined the catalytic degradation activity of WO_3_/F-TiO_2_-{001} for MB, while the photocatalytic activity of benzoquinone decreased slightly, indicating that hydroxyl radical, photo generated holes and superoxide radical all participated in the photo-catalytic degradation of MB. However, photo-generated holes had more effect. According to the literature reports, the hole recombination rate of TiO_2_ is high, while the composite WO_3_/F-TiO_2_-{001} catalyst prepared in this paper hinders the recombination of more holes and photo-generated electrons.

**Fig. 10:**
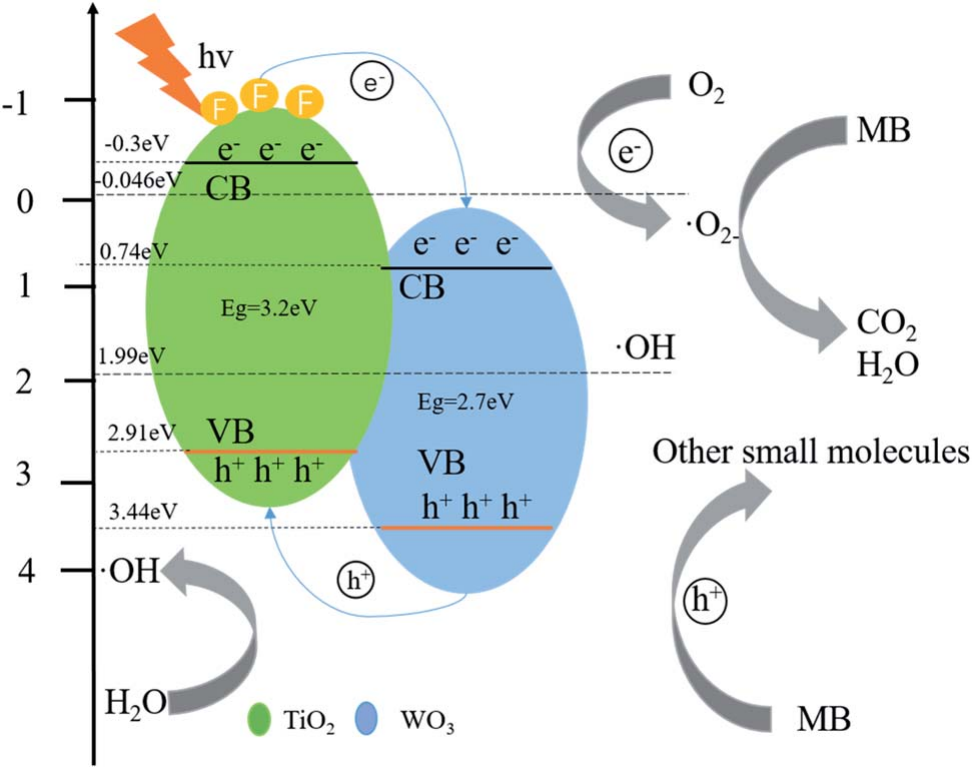
Photocatalytic reaction mechanism of degradation of MB by WO_3_/F-TiO_2_-{001}.

Based on the experimental results and the position of the band gap, we propose a possible mechanism for the better activity of WO_3_/F-TiO_2_-{001} photocatalytic materials. First of all, due to its unique network structure, the specific surface area of the catalyst is greatly increased. The large specific surface area is conducive to the adsorption of reactants and can provide more active sites for the photocatalytic reaction.^72^ In addition, the heterojunction formed by WO_3_ and F-TiO_2_ is a type II heterojunction,^73^ which effectively reduces the recombination efficiency of electrons and holes, and hence electrons and holes have more opportunities to react with O_2_ and H_2_O and generate active materials that can degrade MB. It can be seen from Fig. 10 that the CB potential (−0.3 eV) of anatase TiO_2_ is more negative than the CB potential (0.74 eV) of WO_3_. A strong driving force is generated between the tight non-uniform interfaces, which promotes the transfer of electrons from the CB of TiO_2_ to the CB of WO_3_. Since there are some oxygen vacancies in the material, part of the electrons can be captured. Obviously, the directional transfer of electrons and the trapping of electrons by oxygen vacancies effectively reduce the recombination rate of electron–hole pairs, which is also the main reason for the excellent photocatalytic performance of the photocatalyst. Whereas the CB potential of TiO_2_ (−0.3 eV) is more negative than the potential of O_2_/˙O (−0.046 eV), O_2_ adsorbed on the surface of the photocatalyst is easier to capture electrons on the TiO_2_ CB to form ˙O_2_. This O_2_ can highly oxidize MB and other pollutants. In addition, since the material has some oxygen vacancies, these oxygen vacancies can better adsorb water and play a vital role in the formation of hydroxyl radicals (˙OH). As long as the VB potential of WO_3_ (3.44 eV) is higher than the VB potential of TiO_2_ (2.91 eV), the h^+^ on the WO_3_ VB is easier to transfer to the VB of TiO_2_, and the VB potential of the two photocatalysts is higher than the standard redox potential (1.99 eV) of ˙OH/OH^−^, which makes it easy for the adsorbed water on the surface of the photocatalyst to form strong oxidizing ˙OH. When the active factors such as ˙O_2_^−^ and ˙OH attack, the aromatic ring will be hydroxylated to produce phenolic metabolites. The cationic functional groups of MB molecules may be vertically adsorbed to the surface and eventually produce CO^2^, SO_4_^2−^, NH^4+^ and NO^3−^. Degradation intermediates originated from the initial opening of the central aromatic ring, and the formation of their subsequent metabolites was consistent with the general law of degradation of other complex molecules in water. In addition, it is not negligible that the composite photocatalytic material adsorbs pollutants and the synergy of photocatalysis. The effect makes it have a better degradation effect on MB.

## 4. Conclusion

In this paper, a new type of WO_3_/F-TiO_2_-{001} heterostructure semiconductor material with a three-dimensional network structure was successfully prepared by the hydrothermal method. Compared with WO_3_ and P25, the WO_3_/F-TiO_2_-{001} heterojunction constructed with OH-TiOF_2_ as the precursor exhibits superior photocatalytic performance for the removal of MB induced by visible light. Among them, the FWT5 heterojunction showed higher photocatalytic degradation efficiency. The significant improvement in photocatalytic performance is due to the combination of OH-TiOF_2_ and WO_3_ to form a complex network structure, which provides more photocatalytic area. Meanwhile, the formation of a type II heterojunction enhances the photo-induced charge separation. Therefore, WO_3_/F-TiO_2_-{001} with high photocatalytic efficiency has potential application prospects in the removal of organic pollutants.

## Conflicts of interest

There are no conflicts to declare.

## Supplementary Material
